# Supplementary material for: Promoting Spiritual Coping of Family Caregivers of an Adult Relative with Severe Mental Illness: Development and Test of a Nursing Intervention
Source: Healthcare (Basel). 2024 Jun 22;12(13):1247. doi: 10.3390/healthcare12131247 (PMC11241127; doi:10.3390/healthcare12131247)
Supplement: Supplementary file 1 [file healthcare-12-01247-s001.zip › healthcare-2941924-supplementary.pdf]

Supplement

Table S1 – Individual changes in scores – Brief RCOPE Positive religious/spiritual coping

Participant	Pre-intervention value	Post-intervention value	Difference
1	27	27	=
2	16	19	+3
3	17	18	+1
4	26	26	=
5	13	14	+1
6	14	17	+3
7	23	28	+5
8	22	23	+1
9	11	13	+2
10	13	15	+2

Table S2 – Individual changes in scores – Brief RCOPE Negative religious/spiritual coping

Participant	Pre-intervention value	Post-intervention value	Difference
1	7	7	=
2	10	8	-2
3	8	7	-1
4	6	6	=
5	10	9	-1
6	16	12	-4
7	7	6	-1
8	11	10	-1
9	8	8	=
10	8	8	=

Table S3 – Individual changes in scores – QASCI

Participant	Pre-intervention value	Post-intervention value	Difference
1	46.43	42.86	-3.57
2	51.79	50.00	-1.79
3	37.50	37.50	0
4	53.57	50.00	-3.57
5	39.29	33.93	-5.36
6	23.21	23.21	0
7	46.43	37.50	-8.93
8	62.50	58.93	-3.57
9	57.14	51.79	-5.35
10	53.57	48.21	-5.36

Table S4 – Individual changes in scores – SF-12 – Physical Health Summary

Participant	Pre-intervention value	Post-intervention value	Difference
1	56.37	58.23	1.86
2	52.46	50.87	-1.59
3	57	55.27	-1.73
4	59.44	57.6	-1.84
5	59.8	59.16	-0.64
6	60.11	59.23	-0.88
7	57.89	58.43	0.54

8	54.22	55.81	1.59
9	59.11	57.33	-1.78
10	60.73	59.23	-1.5

Table S5 – Individual changes in scores – SF-12 – Mental Health Summary

Participant	Pre-intervention value	Post-intervention value	Difference
1	53.97	53.24	-0.73
2	52.46	54.95	2.49
3	55.11	57.34	2.23
4	53.65	56.62	2.97
5	54.45	54.73	0.28
6	52.93	54.04	1.11
7	55.96	56.52	0.56
8	53.45	52.02	-1.43
9	50.57	53.28	2.71
10	50.8	54.04	3.24

Table S6 – Summary of Wilcoxon signed rank test results comparing baseline (T0) with post-intervention assessment (T1)

	**Negative Ranks†**			**Positive Ranks‡**			**Test statistics**		
**Outcomes**	*n*	Mean Rank	Sum of ranks	*n*	Mean Rank	Sum of ranks	Ties§	Z	*p*
**Brief RCOPE-PT**									
**Negative Spiritual Coping (T1-T0)**	6	3.50	21.00	0	.00	.00	4	-2.264 [b]	.024*
**Positive Spiritual Coping (T1-T0)**	0	.00	.00	8	4.50	36.00	2	-2.539 [a]	.011*
**SF-12v2**									
**Physical Health Summary (T1-T0)**	7	5.43	38	3	5.67	17.00	0	-1.070[b]	.285
**Mental Health Summary (T1-T0)**	2	4.00	8.00	8	5.88	47.00	0	-1.988 [a]	.047*
**QASCI (T1-T0)**	8	4.5	36.00	0	.00	.00	2	-2.536 [b]	.011*

Brief RCOPE, Brief Scale Spiritual/Religious Coping; SF-12v2, 12-item Short Form Health Survey; QASCI, Brief version of the Informal Caregiver Burden Assessment Questionnaire; * Indicates a statistically significant change; [a] Based on negative ranks; [b] Based on positive ranks; †, Results T1 < Results T0; ‡, Results T1 > Results T0; § Results T1 = Results T0
